# Emerging Role of Chimeric RNAs in Cell Plasticity and Adaptive Evolution of Cancer Cells

**DOI:** 10.3390/cancers13174328

**Published:** 2021-08-27

**Authors:** Sumit Mukherjee, Henry H. Heng, Milana Frenkel-Morgenstern

**Affiliations:** 1 Cancer Genomics and BioComputing of Complex Diseases Lab, Azrieli Faculty of Medicine, Bar-Ilan University, Safed 1311502, Israel; sumit.mukherjee@biu.ac.il; 2 Center for Molecular Medicine and Genetics, Wayne State University School of Medicine, Detroit, MI 48201, USA; hheng@med.wayne.edu; 3 Department of Pathology, Wayne State University School of Medicine, Detroit, MI 48201, USA

**Keywords:** chimeric RNAs, genomic instability, cellular plasticity, cancer evolution

## Abstract

**Simple Summary:**

Fusion of exons or introns from two different genes can lead to the formation of chimeric RNAs. Several recent studies have reported that chimeric RNAs promote tumorigenesis and cancer drug resistance. Therefore, chimeric RNAs are crucial for generating phenotypic diversity between cancer cells that drives the adaptive evolution of cancer. Here, we will discuss the significance of chimeric RNAs in generating functional diversity in cancer cells and their potential impact on developing cancer from an evolutionary viewpoint.

**Abstract:**

Gene fusions can give rise to somatic alterations in cancers. Fusion genes have the potential to create chimeric RNAs, which can generate the phenotypic diversity of cancer cells, and could be associated with novel molecular functions related to cancer cell survival and proliferation. The expression of chimeric RNAs in cancer cells might impact diverse cancer-related functions, including loss of apoptosis and cancer cell plasticity, and promote oncogenesis. Due to their recurrence in cancers and functional association with oncogenic processes, chimeric RNAs are considered biomarkers for cancer diagnosis. Several recent studies demonstrated that chimeric RNAs could lead to the generation of new functionality for the resistance of cancer cells against drug therapy. Therefore, targeting chimeric RNAs in drug resistance cancer could be useful for developing precision medicine. So, understanding the functional impact of chimeric RNAs in cancer cells from an evolutionary perspective will be helpful to elucidate cancer evolution, which could provide a new insight to design more effective therapies for cancer patients in a personalized manner.

## 1. Introduction

Traditionally, cancer development has been accepted as a multistage process driven by the stepwise accumulation of new genetic changes, which promotes the gaining of several abilities that allow cancer cells to survive and proliferate without being subjected to cellular regulatory barriers [1,2,3]. In recent years, however, it has become clear that stochastic cellular macroevolution appears suddenly by saltation for most cancer types, which challenges the neo-Darwinian concept of cancer evolution. Cancer formation requires macroevolution, as only new systems can break a series of barriers from normal tissues/organs/body, where various constraints, including cellular, tissue, and immune factors, prevent the phase transition from a normal cell to cancer [4]. Cancer cells evolve all the way through disease progression, metastasis, and tumor relapse via multiple cycles of two-phased cancer evolution (genome alteration-mediated macroevolution, followed by gene mutation-mediated microevolution) [5,6,7,8]. Cancer evolution is a dynamic process involving genotypic and phenotypic changes, ensuring the high level of plasticity of cancer cells [8,9,10]. Such plasticity, or heterogeneity, is the lifeline for cancer cells, as it helps cancer cells to survive and become dominant under multiple levels of constraints. Constant change is the winning strategy for cancer cells, and genome instability is a powerful mechanism that allows both the survival (by changing genome structure within the macroevolutionary phase) and fitness (by changing gene mutation/epigenetic profile within the microevolutionary phase) of cancer cells [11,12,13,14]. Genomic instability can give rise to gene mutations, chromosomal translocations, alternations of copy number, deletions, and inversions of pieces of DNA [15]. Genomic instability is an important mechanism that enables the acquisition of new characteristics required for oncogenesis, which is the potential driver of cancer evolution [16].

Due to the consequences of genome instability in cancer cells, sometimes two mRNAs can be fused to generate chimeric RNAs [17]. Several recent studies demonstrated that chimeric RNAs are significantly associated with oncogenesis [18,19] and can also promote drug resistance [20,21,22]. The generation of chimeric RNAs could allow cancer cells to switch their functionality. Therefore, chimeric RNAs are an important driver for generating the phenotypic plasticity of cancer cells and increasing their fitness in the tissue environment. Chimeric RNAs could be translated and generate new fusion or chimeric proteins that could alter the normal pathways and lead to cancer development [19,23,24]. Chimeric RNAs could also generate long non-coding RNA (lncRNA), which could regulate cancer cell proliferation [18,25,26]. No study so far has attempted to understand the role of chimeric RNAs in developing and spreading cancers from the perspective of cancer evolution. In this review, we discuss the role of chimeric RNAs in cancer cell plasticity during cancer evolution. Understanding the functional impact of chimeric RNAs through the lens of cancer evolution could be helpful to develop better treatment strategies against cancers.

## 2. Mechanisms of Formation of Chimeric RNAs in Cancer Cells and Their Functional Associations with Cancer Development

**Figure 1:**
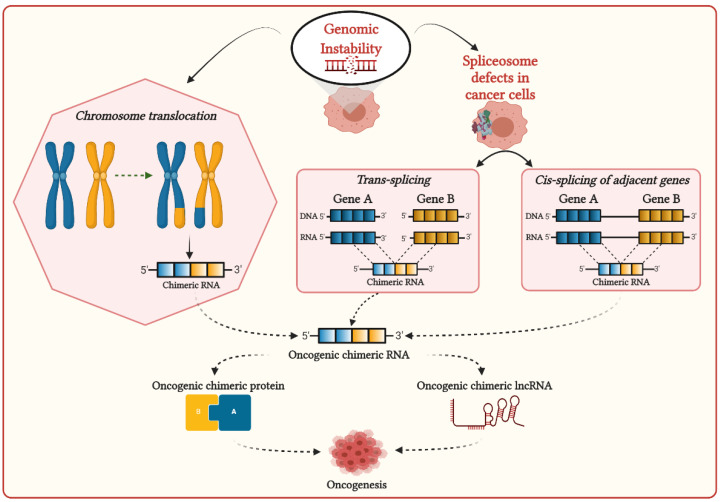
Possible mechanisms for the generation of chimeric transcripts in cancer cells.

Genomic instability can induce chromosomal aberrations such as translocation, enabling the generation of fusion genes, then transcribe them to corresponding chimeric RNAs (Figure 1) [27]. Most chimeric RNAs generated by chromosomal translocation are recurrent and translated in chimeric proteins, which are significantly associated with cancer development [28]. Chimeric RNAs generated by chromosomal aberrations are prevalently observed in hematopoietic malignancies and sarcomas, frequently involving genes required for chromatin regulation and transcriptional control. Chimeric proteins generated by these chimeric RNAs are thought to be the principal driver of oncogenesis, altering chromatin dynamics to activate the oncogene expressions [29]. The well-known recurrent chimeric RNAs generated due to chromosomal aberrations in different cancers, which are associated with cancer-related processes, are listed in Table 1.

The first reported fusion gene BCR-ABL was discovered in human chronic myelogenous leukemia (CML) [48], which is generated by translocation between the q arms of chromosomes 9 and 22 and is denoted as t(9;22). Fusion protein produced from this BCR-ABL chimeric transcript altered constitutively active ABL1 kinase that can promote the development of CML [30]. Another chromosomal translocation t(15;17) was detected in acute promyelocytic leukemia (APL), resulting in the formation of promyelocytic leukemia–retinoic acid receptor α (PML-RARα) fusion oncoprotein that can interplay with retinoic X receptors (RXR) and promote the deregulation of epigenetic modifications [31,32,33]. A recurrent gene fusion TMPRSS2-ERG was observed in more than fifty percent of prostate cancer cases with the deletion of del(q22) and t(7;21)(1,26–28), resulting in translocation of the ERG gene (21q22.3) or the ETV1 gene (7p21.2) to the TMPRSS2 gene (21q22.2) promoter region [44,45]. This fusion leads to the overexpression of the oncogene ERG or ETS transcription factors in response to androgens induced by the TMPRSS2 promoter, which promotes the generation of molecular heterogeneity and the formation of high-grade tumors [33,49,50]. In Burkitt lymphoma, three translocations t(8;14)(q24;q32), t(2;8)(p11;q24), or t(8;22)(q24;q11) were observed, where, in all cases, the breakpoint in chromosome 8 is within the MYC gene, and the other breakpoint is within an immunoglobulin gene [51,52]. These translocations promote the MYC gene to become continuously expressed due to the impact of regulatory elements of the immunoglobulin genes, which are crucial for initiating oncogenesis [27]. Several fusions associated with the MLL1 gene were found in acute leukemia, which was generated due to recurrent chromosomal rearrangements involving 11q23 [53,54,55,56]. MLL1 fusion-positive leukemia has a remarkably low somatic mutation rate, suggesting that MLL1 fusions are the potential drivers of cancer development [56,57,58]. Altogether, it can be suggested that the appearance of chimeric RNAs due to chromosomal translocation promotes phenotypic plasticity for cancer development.

The interplay of splicing factors and RNA-binding proteins (RBPs) are found to play an important role in DNA–RNA hybrid (R-loop) formation during transcription to prevent RNA-induced genome instability [59]. In cancers, mutations in the spliceosome machinery can affect R-loop formation, promoting genomic instability [60,61,62]. Genome instability and mutations in spliceosome machinery can stimulate aberrant splicing, including cis- and trans-splicing, leading to the generation of chimeric RNAs in cancer cells (Figure 1) [63]. Cis-splicing is the mechanism by which two neighboring genes in the same strands are transcribed in the same orientation and generate read-through chimeric transcripts [64]. The recurrent read-through chimeric transcripts generated via cis-splicing were prevalently observed in renal carcinoma [65], prostate cancer [66], and breast cancer [67]. In prostate cancer, SLC45A3–ELK4 is the most common chimeric RNA generated by cis-splicing [68,69]. This SLC45A3-ELK4 acts as lncRNA and regulates the proliferation of prostate cancer cells [25]. This fusion is recurrent and regarded as a potential biomarker for prostate cancer. Another method for generating chimeric RNAs is trans-splicing, by which two individual pre-mRNA molecules can be fused. Although trans-splicing is considered a noncanonical splicing process in humans, recent studies demonstrated that trans-splicing could be involved in generating chimeric RNAs in human cells. The two most common examples are JAZF1–SUZ12 [70,71] in endometrial stromal tumors and PAX3–FOXO1 [72] in rhabdomyosarcoma, where, in both cases, identical chimeric RNAs were found as chromosomal translocation from cancer cells and RNA trans-splicing from normal human cells. Therefore, the generation of identical chimeric RNAs in cancer cells by chromosomal translocation, which is also generated in normal cells due to different mechanisms, could potentially be associated with distinct pathological consequences in cancers.

## 3. Functional Impact of Chimeric RNAs in Cancer Heterogeneity and Drug Resistance

**Figure 2:**
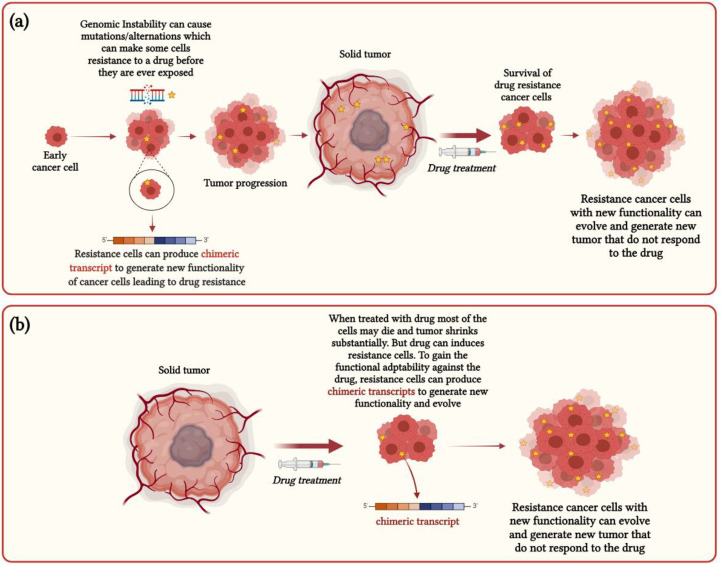
Functional impact of chimeric RNAs in cancer heterogeneity and drug resistance: (**a**) functional model for chimeric RNAs generated at the early stage of cancers; (**b**) functional model for chimeric RNAs generated at the later stage of cancers.

Chimeric RNAs could be generated at the beginning of tumor development due to genomic instability that can sometimes generate novel functionality in cancer cells, enabling them to resist specific drugs before they are ever exposed (Figure 2a), by which some drug treatments might not work for cancer patients. For example, in acute and chronic leukemias and non-Hodgkin’s lymphoma, fusion tyrosine kinases (FTKs) such as BCR–ABL, TEL–ABL, TEL–JAK2, TEL–PDGFβR, TEL–TRKC(L), and NPM–ALK are generated by chromosomal translocations [73,74,75]. In addition, FTK-transformed cells exhibit resistance against cytostatic drugs such as cisplatin and mitomycin C [73]. Therefore, these FTK-transformed cells are protected from drug-mediated DNA damage, which ensures the survival of cancer cells in response to drug treatment. Further, oncogenic BRAF fusions are frequently identified in melanomas prior to drug treatment, and BRAF fusion-positive cancers show resistance to BRAF inhibitor drugs such as vemurafenib and dabrafenib [76,77].

Chimeric RNAs could arise in the later stage of cancers after drug treatments that induce cancer cells’ resistance specific to this drug and can drive the adaptive evolution of drug resistance cancer cells (Figure 2b). For example, in non-small-cell lung carcinoma (NSCLC), EGFR tyrosine kinase inhibitors (EGFR-TKI) are used as treatment, but in some cases, EGFR-TKI resistance is detected within one year of drug administration [78]. A recent case study reported that a 72-year-old male lung adenocarcinoma patient with an EGFR deletion mutation initially responded to EGFR-TKI treatment but later developed acquired resistance against EGFR-TKI. Subsequently, a new fusion KIF5B-RET was identified from the repeated liquid biopsy samples from the post-treatment, suggesting that the emergence of this KIF5B-RET chimeric RNAs could potentially be associated with the EGFR-TKI drug resistance [78]. Further, several chimeric RNAs such as LDLR-RPL31P11, VCL-ADK, TAF15-AP2B1, and MYH9-EIF3D were detected from the RNA-seq data of the docetaxel-resistant prostate cancer cell lines, which are not found to be associated with primary resistance and thought to be a crucial driver for acquired resistance [79]. The occurrence of different fusions involving the ABCB1 gene, which encodes multidrug resistance protein 1 (MDR1), was observed in post-treatment high-grade serous ovarian cancer (HGSC) and end-stage breast cancer samples [80]. The appearance of these recurrent fusions after chemotherapy treatments indicates that they could propel the positive selection for MDR1 expression and play an important role in the functional adaptation of drug-resistant cancer cells [80].

## 4. Chimeric RNAs Are the Essential Driver for Generating Phenotypic Diversity in Cancer Cells

Identifying a vast number of cancer-specific chimeric RNAs from the pan-cancer analysis of whole-genome (PCAWG) projects [81,82] of more than 2600 tumor samples from different cancers opened questions regarding the functional relevance of these chimeric RNAs. So, now, the most intriguing argument is that chimeric RNAs generated in cancer cells are non-functional, or they can contribute to functional diversity in cancer cells. Recent evidence suggested that cancer cells adapt to cope with the stress associated with high levels of transcription [83,84,85]. So, it makes sense that the production of different transcripts, including chimeric transcripts, could generate functional diversity that helps cancer cells adapt to stress conditions. Moreover, from an evolutionary perspective, generating a vast number of non-functional transcripts could increase the bioenergetic cost of the system, which can reduce the fitness of the cells to a new environment. Therefore, the generation of various chimeric transcripts at the different stages of cancer depends on cancer-related stress unique to individuals. These chimeric RNAs are important for cancer cells to alter their functionality in such a way so that they can survive and proliferate in different stress conditions. Further, recent evidence supported that recurrent chimeric RNAs across different cancer types have functional relevance in oncogenesis and cancer cell proliferation and might be the driver for some cancers [17,18]. These recurrent cancer-specific chimeric RNAs could be translated to generate novel proteins or act as lncRNAs, contributing to new functionality and a regulatory role in cancer cells [18]. Novel chimeric proteins generated from chimeric RNAs help the survival of cancer cells and adapt by regulating the dynamics of protein interaction networks [23,24]. For example, they can activate cell growth pathways (by functioning as oncoproteins), switch pathways, impact normal protein interaction patterns, and increase heterogeneity, which is essential for cancer evolution [24,86]. Additionally, based on the several lines of evidence discussed earlier, we suggest that the appearance of chimeric RNAs in cancer cells could also increase the functional expansion of cancer cells to survive in the face of drug-mediated damage. Therefore, from the standpoint of cancer evolution, the appearance of chimeric RNAs could be beneficial for the system, generating phenotypic plasticity in cancer cells. This could drive the fitness of cancer cells and support Darwinian evolution.

## 5. How Could Chimeric RNAs Lead to Cancer Evolution?

Chimeric RNAs are evidently involved in cancer [17,18,87,88]. In cancer cells, chimeric RNAs could either produce oncogenic chimeric protein or oncogenic lncRNA, impacting cancer evolution and heterogeneity [25,64,86,89,90,91]. Often, recurrent chimeric RNAs are directly associated with oncogenesis and cellular proliferation. In contrast, most nonrecurrent chimeric RNAs likely contribute to cancer evolution by promoting heterogeneity. This may be the reason some have suggested that many chimeric RNAs generated in cancer cells are merely transcriptional noise. However, it should be noted that the concept of biological “noise” is fundamentally different from the physical concept where the “noise” (or errors in measurement) should be eliminated. In biology, noise is system heterogeneity instead, which not only represents unique biological information or informational context but can also play an essential role in evolution [92,93,94,95,96]. Some non-functional RNAs are eventually degraded, with no direct role in cellular function, including chimeric RNAs. However, under different selection conditions, the role of “signals” and “noise” can switch, which is the basis for heterogeneity-mediated evolutionary selection. In addition, a high “noise” background can favor the function of the strongest signals (thus reducing the impact of less dominant players, which increases specificity). In recent years, multiple levels of heterogeneity have been observed from normal tissue, especially in cancer, urgently requiring us to rethink the concept of “noise” [8,97]. It is known that an increased level of “noise” in the transcriptome favors cancer evolution [98,99,100]. Under high-stress conditions, there often are transitions from noise to a recurrent pattern as a result of evolutionary selection.

Recently, the concept of fuzzy inheritance was proposed as a common mechanism of genomic and nongenomic heterogeneity [8]. According to the Genome Architecture Theory, the limited predictability between genotype and phenotype, also called missing heritability, can be explained by a less determined function of gene coding. In contrast to the classic genetic viewpoint, each gene can code an array of phenotypes on a sliding scale rather than two phenotypes being defined by Mendelian binary categories (dominant vs. recessive). The environment selects a given phenotype within the potential coded range. Thus, when passing on genomic information to the next generation, the genomic package codes an array of potential phenotype states, rather than the exact phenotype of parents, that is transferred [8]. Interestingly, the formation of chimeric RNA without chromosomal translocation represents yet another mechanism of fuzzy inheritance.

From a biocode point of view, the production of such chimeric RNA is likely regulated by a new organic code [97]. When under the condition of genome instability and other factors yet to be identified, the dynamics of the spliceosome increase, promoting the formation of chimeric RNAs and resulting in increased fuzzy inheritance. In this case, cells favor the creation and/or modification of information mediated by chimeric RNAs. By this logic, chimeric RNAs should not only be explained as spliceosome defects but instead as a positive contribution from an altered splicing process. This explanation is consistent with the phenomenon that chimeric RNAs can be more frequently detected from cancer cells, as cancer is a new emergent system that must break multiple levels of constraints in cellular and tissue environments, and the combination of heterogeneity, aided by the creation of new information by chimeric RNAs, is the ultimate material needed for evolution to work.

Interestingly, there might be a functional difference between different types of chimeric RNAs. For those that result from chromosomal translocations, there may be more significant phenotypes; in addition to specific chimeric RNA-mediated information, the altered genome will likely generate new genome-level information when the karyotype coding is altered by translocation. In the case of CML, for example, additional chromosomal alterations could interfere with treatment response. Meanwhile, chimeric RNAs without chromosomal translocations are more likely involved in the microevolutionary phase, similar to many cancer genes.

**Figure 3:**
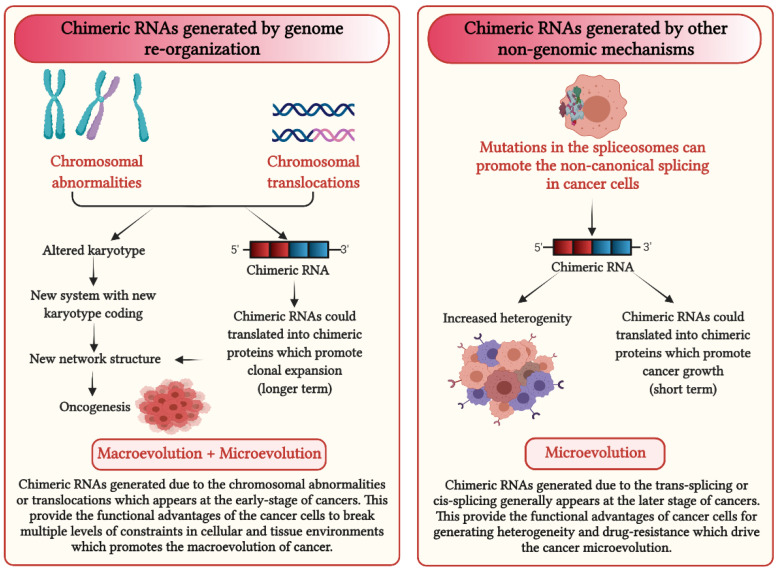
Impact of chimeric RNAs on cancer evolution.

In recent years, as increasing numbers of cancer genomes were sequenced, the concept of cancer evolution has drastically been changing [101]. The lack of stepwise accumulation of cancer gene mutations and overwhelming chromosomal aberrations displays the highly dynamic pattern of cancer evolution [102,103]. To explain the different patterns of cancer evolution, a two-phased cancer evolutionary model was proposed [8]. Specifically, the genome reorganization-mediated punctuated phase and gene mutation-mediated stepwise phase are separated. In other words, macroevolutionary transition is often achieved by new karyotype formation punctually, while microevolutionary transition often is helped by specific genes to grow the cellular population gradually. Currently, there are many examples of specific chimeric RNAs contributing to cancer evolution and the phenomenon of drug resistance. Based on the two-phased cancer evolutionary model, many of these chimeric RNAs likely play a role within the microevolutionary phase (Figure 3). It is interesting to investigate, for example, whether harsher treatment could promote the generation of chimeric RNAs, which would help drug resistance, as it is known that harsh treatments can promote genome chaos [104]. It is also possible that stabilizing the spliceosome could reduce the opportunity to produce chimeric RNAs. Like any new research topic, there always are more questions than answers.

## 6. Conclusions and Future Perspective

From the beginning of the discovery of chimeric RNAs, they have been found to be associated with cancers. With the advancement of high-throughput sequencing technology and cancer genomics, several chimeric RNAs have been identified in different cancer samples, where some chimeric RNAs were found recurrent and some specific to the sample. As discussed above, several chimeric RNAs were found to be associated with oncogenesis, and several were reported to enable drug resistance in tumors, which supports their clinical significance for targeting them to design new cancer treatments. Further, chimeric proteins generated from these chimeric RNAs have different structures from their parental proteins, which helps them to alter the protein interaction networks in cancer cells that enable their survival and proliferation. Therefore, designing drugs that could target chimeric proteins could be helpful in cancer therapy. Hence, understanding the appearance of chimeric RNAs could guide the direction of cancer evolution, which could be useful to the development of a new strategy for cancer treatment.

As cancer is an evolutionary process, cancer cells should undergo adaptive evolution and create phenotypic diversity to fit into a new environment. The generation of chimeric RNAs is associated with the functional expansion of cancer cells and the creation of phenotypic diversity. Selection directly acts on phenotypes instead of genotypes, and overall phenotypic traits of the cell could determine its perseverance and fate in a cell population. Therefore, the production of chimeric RNAs is important for generating the phenotypic plasticity of cancer cells that can provide a fitness advantage to adapt to new environmental conditions. However, there are several open questions regarding (1) how selection can choose partner genes for chimeric RNAs, (2) how chimeric RNAs can contribute to the fitness of cancer cells, (3) what the mechanism for drug resistance by generating chimeric RNAs is, (4) what the evolutionary principles for some chimeric RNAs are recurrent, and (5) what the fate of chimeric RNAs in cancer cells is. Potential solutions to each of these questions need the understanding of chimeric RNAs through the lens of evolution with the communication of clinical studies. This could help to decipher cancer evolution, which could facilitate the improvement of personalized treatment strategies.

## Figures and Tables

**Table 1:** Chimeric RNAs generated due to chromosomal aberrations in different cancers and their potential functions.

Chimeric RNA	Gene 1	Gene 2	Associated Cancers	Potential Function	Chromosomal Aberrations
BCR-ABL	BCR	ABL	Chronic myelogenous leukemia (CML)	BCR-ABL fusion protein alters constitutively active ABL1 kinase and activates a variety of signaling pathways that promote CML development [30]	Translocation t(9;22)
PML-RARα	PML	RARα	Acute promyelocytic leukemia (APL)	PML-RARα fusion protein interplays with retinoic X receptors (RXR) and promotes the deregulation of epigenetic modifications [31,32,33]	Translocation t(15;17)
RUNX1–RUNX1T1	RUNX1	RUNX1T1	Acute promyelocytic leukemia (APL)	RUNX1–RUNX1T1 fusion protein interacts with other proteins to repress transcription and induce leukemogenesis in myeloid progenitor cells [34]	Translocation t(8;21)
EWS–FLI1	EWS	FLI1	Ewing’s sarcoma (EWS)	EWS–FLI1 fusion transcription factors upregulate genes associated with the cell cycle, invasion, and proliferation pathways [35]	Translocation t(11;22)
EWS–ERG	EWS	ERG	Ewing’s sarcoma (EWS)	EWS–ERG fusion transcription factors upregulate genes associated with the cell cycle, invasion, and proliferation pathways [36]	Translocation t(21;22)
PAX8-PPARγ1	PAX8	PPARγ1	Thyroid follicular carcinomas	PAX8-PPARγ1 fusion protein can act as a dominant-negative inhibitor of wild-type PPARγ and can activate or repress PAX8-responsive genes [37]	Translocation t(2;3)(q13;p25)
SS18–SSX1	SS18	SSX1	Synovial sarcoma	SS18-SSX1 fusion protein employs core Wnt pathway transcription factors to induce Wnt target gene expression in synovial sarcoma [38]	Translocation t(X;18)
SS18–SSX2	SS18	SSX2	Synovial sarcoma	SS18-SSX2 fusion protein induces epigenetic gene deregulation and promotes the development of synovial sarcoma [39]	Translocation t(X;18)
MYB-NFIB	MYB	NFIB	Adenoid cystic carcinomas (ACC)	MYB-NFIB fusion proteins promote the upregulation of MYB, which can drive the development of adenoid cystic carcinoma (ACC) [40,41]	Translocation t(6;9)(q22–23;p23–24)
MECT1-MAML2	MECT1	MAML2	Mucoepidermoid carcinoma	MECT1-MAML2 fusion protein undermine two signaling pathways, CREB and Notch, that could be potentially important in cancer development [42,43]	Translocation t(11;19)(q14–21;p12–13)
TMPRSS2-ERG	TMPRSS2	ERG	Prostate cancer	Expression of TMPRSS2-ERG chimeric transcripts induces overexpression of the transcription factor ERG, which promotes invasion in human prostate cancer development [44,45]	Del(q22) and Translocation t(7;21)(1,26–28)
EML4–ALK	EML4	ALK	Non-small-cell lung cancer (NSCLC)	Generation of this EML4–ALK chimeric RNA leads cancer transformation by activating downstream reactions in the ALK signaling pathway [46]	Inversion of chromosome 2 (inv2) (p21:p23)
PVT1–MYC	PVT1	MYC	Medulloblastoma	Chromothripsis in medulloblastoma promotes the recurrent translocations, which enable the fusion of lncRNA PVT1 to MYC gene as a consequence of the continuous oncogenic effect via MYC amplification [47]	Chromothripsis
